# Neonatal deaths in rural Karnataka, India 2014–2018: a prospective population-based observational study in a low-resource setting

**DOI:** 10.1186/s12978-020-01014-6

**Published:** 2020-11-30

**Authors:** Sangappa M. Dhaded, Manjunath S. Somannavar, Janet L. Moore, Elizabeth M. McClure, Sunil S. Vernekar, S. Yogeshkumar, Avinash Kavi, Umesh Y. Ramadurg, Tracy L. Nolen, Robert L. Goldenberg, Richard J. Derman, Shivaprasad S. Goudar

**Affiliations:** 1 Women’s and Children’s Health Research Unit JN Medical College, KLE Academy of Higher Education and Research Belagavi, Belagavi, Karnataka India; 2 RTI International, Durham, NC USA; 3 S Nijalingappa Medical College and HSK Hospital Bagalkot, Bagalkot, Karnataka India; 4 Columbia University, New York, NY USA; 5 Thomas Jefferson University, Philadelphia, PA USA

**Keywords:** Neonatal death, Cause of death, India

## Abstract

**Background:**

Neonatal mortality causes a substantial proportion of the under-5 mortality in low and middle-income countries (LMIC).

**Methods:**

We undertook a prospective, population-based research study of pregnant women residing in defined geographic areas in the Karnataka State of India, a research site of the Global Network for Women’s and Children’s Health Research. Study staff collected demographic and health care characteristics on eligible women enrolled with neonatal outcomes obtained at delivery and day 28. Cause of neonatal mortality at day 28 was assigned by algorithm using prospectively defined variables.

**Results:**

From 2014 to 2018, the neonatal mortality rate was 24.5 per 1,000 live births. The cause of the 28-day neonatal deaths was attributed to prematurity (27.9%), birth asphyxia (25.1%), infection (23.7%) and congenital anomalies (18.4%). Four or more antenatal care (ANC) visits was associated with a lower risk of neonatal death compared to fewer ANC visits. In the adjusted model, compared to liveborn infants ≥ 2500 g, infants born weighing < 1000 g RR for mortality was 25.6 (95%CI 18.3, 36.0), for 1000-1499 g infants the RR was 19.8 (95% CI 14.2, 27.5) and for 1500–2499 g infants the RR was 3.1 (95% CI 2.7, 3.6). However, more than one-third (36.8%) of the deaths occurred among infants with a birthweight ≥ 2500 g. Infants born preterm (< 37 weeks) were also at higher risk for 28-day mortality (RR 7.9, 95% CI 6.9, 9.0) compared to infants ≥ 37 weeks. A one-week decrease in gestational age at delivery was associated with a higher risk of mortality with a RR of 1.3 (95% CI 1.3, 1.3). More than 70% of all the deliveries occurred at a hospital. Among infants who died, 50.3% of the infants had received bag/mask ventilation, 47.3% received antibiotics, and 55.6% received oxygen.

**Conclusions:**

Consistent with prior research, the study found that infants who were preterm and low-birth weight remained at highest risk for 28-day neonatal mortality in India. Although most of births now occur within health facilities, a substantial proportion are not receiving basic life-saving interventions. Further efforts to understand the impact of care on infant outcomes are needed.

*Study registration* The trial is registered at clinicaltrials.gov. ClinicalTrial.gov Trial Registration: NCT01073475

## Background

Worldwide, about 2.8 million babies die each year before the completion of one month of life. India contributes to quarter of these deaths [1, 2]. In India alone, almost 0.7 million neonatal deaths were estimated to occur in 2015 [3]. Many of these neonatal deaths are believed to occur because of potentially preventable causes such as complications of preterm birth, infectious disease and asphyxia [4]. These three causes are estimated to be responsible for almost 84% of the deaths [5].

World-wide, many of the neonatal deaths occur at home and because of lack of accurate vital registration systems, the current global mortality estimates have limitations. In low and middle-income countries (LMIC) such as India, the estimates may under-represent the true burden and be inaccurate [6]. Clinician-assigned cause of death, which is the most common method used, may be inaccurate for several reasons, including lack of diagnostic tools such as autopsies, placental histology, X-rays, as well as lack of routine bacterial cultures [6, 7]. We have previously demonstrated that the Global Network Cause of Death Algorithm can be used to classify causes of neonatal deaths across low-resource settings such as India [8, 9]. Moreover, there are limited data regarding representative rural, population-based Indian data of causes and risk factors of neonatal mortality. Earlier Indian studies had relatively smaller sample sizes [10–12]. This study aimed to identify causes and risk factors of neonatal deaths in rural Belagavi from 2014 to 2018.

## Methods

This study was conducted as part of the Global Network for Women’s and Children’s Health Research (Global Network)’s Maternal Newborn Health Registry (MNHR), a population based, observational study conducted in six low-resource countries, including India [13, 14]. The objective of the MNHR is to enrol all pregnant women residing within defined geographic areas, study clusters, which generally have 300 to 500 deliveries per year [15]. This analysis includes data collected from pregnant women enrolled in the Belagavi site’s MNHR clusters from 2014 to 2018.

All pregnant women residing within a study cluster, or giving birth within the cluster, were approached as early as possible during their pregnancy for inclusion in the MNHR. Following informed consent, women were followed by trained study staff, known as registry administrators (RAs). The RAs enrolled consenting pregnant women and completed perinatal outcome forms for each woman enrolled in the MNHR through 42 days postpartum. RAs collected information on prenatal services and the health status of the mother, including age, weight, height and educational status. Pregnancy outcomes, neonatal interventions and treatment received were also recorded.

The RAs also completed perinatal cause of death evaluation form if the baby died within 28 days of life. The cause of death questionnaire was completed by the staff interviewing the mother, family and health care providers after the death occurred, within two weeks after death. When available, we included hospital-based information from review of clinical records. The detailed methodology for assignment of cause of death is published elsewhere [9]. Briefly, the algorithm first identified if a major congenital anomaly was present. Infection is next determined to be the cause of death if there was no major congenital anomaly and an infection was present or suspected, such as tetanus, omphalitis, sepsis or pneumonia. In absence of both anomaly and infection, then the cause was assigned based on the gestational age at birth. Asphyxia was assigned as the cause of death in term infants if the baby had signs of breathing difficulty. Among term infants, if no signs of difficulty in breathing at birth or respiratory distress were present, the cause of death was assigned as unknown. For preterm infants between 34 and 37 weeks (or 2000–2500 g), asphyxia was assigned as the cause if the neonate had breathing difficulties and/or the mother experienced any complications of pregnancy. If the infant was < 34 weeks and/or < 2000 g and none of the above conditions were present, the cause of death was assigned as complications of prematurity.

### Statistical analyses

All study data were reviewed and cleaned by research staff and then entered into a local secure study computer where edits were performed. Data were then transmitted to a central data coordinating center (RTI International) where additional edits were performed and then resolved by the site.

Generalized linear models were used to evaluate the relationship of potential risk factors with neonatal death < 28 days. Relative risks, 95% confidence intervals and p-values were obtained from log Binomial models as a function of each individual maternal or neonatal characteristic using generalized estimating equations to account for the correlation of outcomes within cluster. A predictive model for neonatal mortality < 28 days was developed using forward selection of maternal and neonatal characteristics associated with 28-day neonatal mortality based on the quasi-likelihood under the Independence Model Criterion (QIC) to evaluate model fit. Relative risks, 95% confidence intervals and p-values were obtained from the resulting predictive multivariable Poisson model using generalized linear model using generalized estimating equations to account for the correlation of outcomes within cluster [16].

## Results

**Fig. 1:**
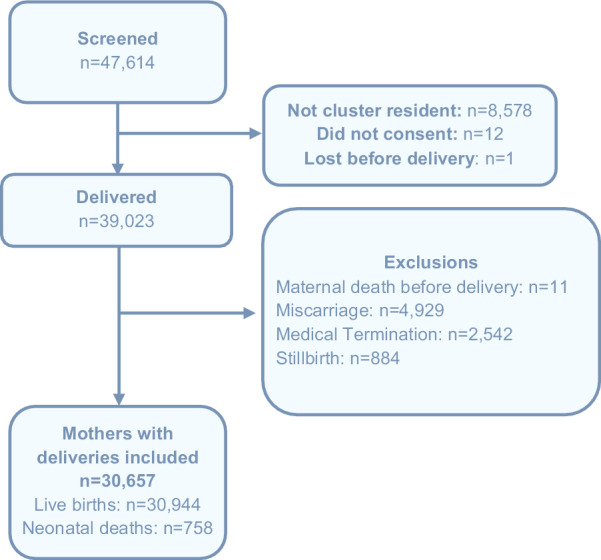
Enrollment diagram

From 2014 to 2018, 47,614 women were screened (Fig. 1). Of these, 99% were enrolled in the study. We excluded women who did not reside within a study cluster (N = 8578), those who died prior to delivery (N = 11), and births that resulted in a miscarriage (N = 4929), a medically terminated pregnancy (N = 2542) or a stillbirth (N = 884). In this analysis, 30,657 women and their 30,944 newborns were included, of which there were 758 neonatal deaths. Of these, 8 were missing the cause of death form and were excluded, resulting in a total of 750 neonatal deaths in the cause of death analysis.

**Fig. 2:**
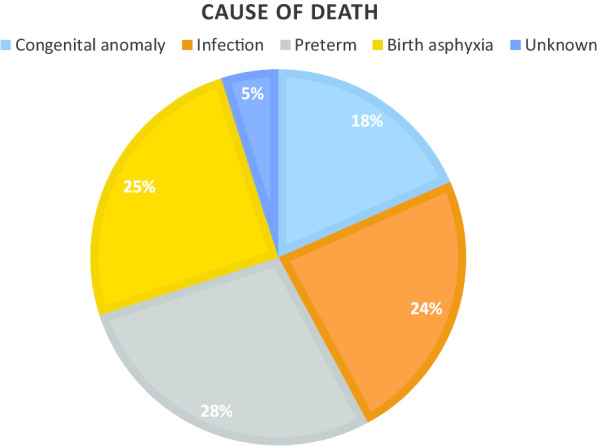
Cause of 28-day neonatal mortality in Belagavi, India 2014–2018

From 2014 to 2018, the neonatal mortality rate was 24.5 per 1000 live births (Table 1). This rate was highest in 2014 (28.6 per 1000) and lowest in 2018 (16.8 per 1000). Overall, the majority of deaths were attributed to complications of preterm birth (27.9%), birth asphyxia (25.1%), followed by infection (23.7%) and congenital anomalies (18.4%) (Fig. 2). In 2014, the highest proportion of deaths (33.7%) were attributed to infection followed by asphyxia (22.8%), while 20.2% were attributed to congenital anomalies. While in 2018, prematurity attributed deaths (34.8%) were more common followed by asphyxia (29.2%) and infection (15.7%).

**Table 1:** 28-day neonatal mortality by year and cause of death 2014–2018

	2014	2015	2016	2017	2018	Overall
Live births, N	6789	6623	6219	5774	5539	30,944
Neonatal mortality < 28 days, N (Rate/1000)	194 (28.6)	167 (25.2)	153 (24.6)	151 (26.2)	93 (16.8)	758 (24.5)
Cause of death, N (%)	193	167	152	149	89	750
Congenital anomaly	39 (20.2)	31 (18.6)	25 (16.4)	31 (20.8)	12 (13.5)	138 (18.4)
Infection	65 (33.7)	45 (26.9)	25 (16.4)	29 (19.5)	14 (15.7)	178 (23.7)
Prematurity	36 (18.7)	44 (26.3)	57 (37.5)	41 (27.5)	31 (34.8)	209 (27.9)
Asphyxia	44 (22.8)	41 (24.6)	40 (26.3)	37 (24.8)	26 (29.2)	188 (25.1)
Unknown	9 (4.7)	6 (3.6)	5 (3.3)	11 (7.4)	6 (6.7)	37 (4.9)

Table 2 shows the delivery location and birth attendant by infant status at day 28. In both groups, more than half of the deliveries occurred with an obstetrician present and more than 70% of the deliveries occurred at a hospital.

**Table 2:** Delivery attendant and location by neonatal status at day 28

	Neonatal mortality < 28 Days	Alive at 28 days
Live births, N	758	30,185
Delivery attendant, N (%)	757	30,181
Obstetrician	428 (56.5)	15,192 (50.3)
Non-OB physician	95 (12.5)	3,212 (10.6)
Nurse/nurse midwife	189 (25.0)	10,996 (36.4)
Traditional birth attendant	4 (0.5)	102 (0.3)
Family	26 (3.4)	589 (2.0)
Self-delivery	14 (1.8)	81 (0.3)
Other	1 (0.1)	9 (0.0)
Delivery location, N (%)	757	30,182
Hospital	568 (75.0)	21,607 (71.6)
Clinic/health center	133 (17.6)	7,485 (24.8)
Home	39 (5.2)	753 (2.5)
Other	17 (2.2)	337 (1.1)

Next, we examined maternal characteristics of the infants alive at day 28 compared to those who died by day 28 (Table 3). Maternal age was not associated with whether the infant survived to day 28 or not. Women with no education (RR 2.13, 95% CI 1.40, 3.26) or only primary or secondary school educations (RR 1.51, 95% CI 1.07, 2.13) had a higher risk of neonatal death compared to women with a university education. The distribution of body-mass-index (BMI) at first antenatal care visit (ANC) was similar between the women with and without a neonatal death with RR of 0.95 (95% CI, 0.81, 1.11) and 1.09 (95% CI, 0.86, 1.39) for underweight and overweight categories respectively. Both nulliparous women (RR 1.30, 95% CI, 1.08, 1.56) and women with more than 2 prior pregnancies (RR 1.54, 95% CI 1.16, 2.04) had a greater risk of a neonatal death. Women who had received 4 or more ANC visits had a lower risk of neonatal death compared to all groups with fewer ANC visits; however, the gestational age at the enrolment, which corresponded to the ANC first visit, was not significantly associated with risk.

**Table 3:** Maternal characteristics: infants alive at day 28 vs. those with a neonatal death

	Neonatal Mortality < 28 Days	Alive at 28 Days	RR (95% CI)^b^	Wald p-value^b^
Maternal age, N (%)	758	30,185		0.5303
< 20	104 (13.7)	3842 (12.7)	1.12 (0.90, 1.41)	0.3075
20–25	487 (64.2)	20,299 (67.2)	Reference	–
26–30	145 (19.1)	5309 (17.6)	1.13 (0.94, 1.38)	0.2000
> 30	22 (2.9)	735 (2.4)	1.24 (0.83, 1.85)	0.2935
Maternal education, N (%)	758	30,183		0.0012
No formal education	126 (16.6)	3555 (11.8)	2.13 (1.40, 3.26)	0.0005
Primary/Secondary	580 (76.5)	23,434 (77.6)	1.51 (1.07, 2.13)	0.0196
University +	52 (6.9)	3194 (10.6)	Reference	–
Body Mass Index, N (%)	756	30,165		0.5449
Underweight (< 18.5)	250 (33.1)	10,406 (34.5)	0.95 (0.81, 1.11)	0.5132
Normal (18.5–24.9)	447 (59.1)	17,633 (58.5)	Reference	–
Overweight (≥ 25)	59 (7.8)	2126 (7.0)	1.09 (0.86, 1.39)	0.4751
Parity, N (%)	758	30,183		0.0003
0	329 (43.4)	11,585 (38.4)	1.30 (1.08, 1.56)	0.0060
1–2	360 (47.5)	16,561 (54.9)	Reference	–
≥ 3	69 (9.1)	2037 (6.7)	1.54 (1.16, 2.04)	0.0026
Number of antenatal care visits, N (%)	758	30,183		< .0001
0–1	21 (2.8)	127 (0.4)	9.11 (6.16, 13.45)	< .0001
2	108 (14.2)	914 (3.0)	6.77 (4.75, 9.65)	< .0001
3	218 (28.8)	6152 (20.4)	2.26 (1.86, 2.74)	< .0001
≥ 4	411 (54.2)	22,990 (76.2)	Reference	–
Gestational age at enrollment, N (%)	756	30,091		0.2470
< 8 weeks	283 (37.4)	10,892 (36.2)	Reference	–
8.0–11.6 weeks	247 (32.7)	11,006 (36.6)	0.87 (0.74, 1.01)	0.0686
12.0–20.0 weeks	177 (23.4)	6422 (21.3)	1.06 (0.85, 1.32)	0.5987
> 20,0 weeks	49 (6.5)	1771 (5.9)	1.07 (0.78, 1.47)	0.6814

^a^For multiple pregnancies the same maternal information is repeated for each infant

^b^Relative risks and p-values are obtained from log Binomial models a function of each individual maternal characteristic using generalized estimating equations to account for the correlation of outcomes within cluster

We next evaluated the infant characteristics (Table 4). Infants born weighing < 2500 g had a substantially higher risk of 28-day mortality, with the highest risk among the lowest birth weight categories, compared to those ≥ 2500 g. Compared to liveborn infants ≥ 2500 g, the 28-day neonatal mortality risks were as follows: for infants born weighing < 1000 g, the relative risk (RR) was 82.6 (95% CI 75.1, 90.9); for those born weighing 1000–1499 g, the RR was 41.1 (95% CI 33.9, 49.9); and for 1500–2499 g infants, the RR was 3.7 (95% CI 3.2, 4.3). While 80.1% of infants weighed ≥ 2500 g at birth, numerically, the largest proportion of neonatal deaths (36.8%) also occurred among infants in the ≥ 2500 g birthweight category.

**Table 4:** Neonatal characteristics, interventions and treatment by neonatal outcome at 28 days

	Neonatal mortality < 28 Days	Alive at 28 Days	RR (95% CI)^b^	Wald p-value^b^
Birth weight, N (%)	750	30,184		< .0001
< 1000 g	108 (14.4)	8 (0.0)	82.63 (75.10, 90.91)	< .0001
1000-1499 g	112 (14.9)	130 (0.4)	41.12 (33.90, 49.87)	< .0001
1500-2499 g	254 (33.9)	5872 (19.5)	3.68 (3.17, 4.26)	< .0001
≥ 2500 g	276 (36.8)	24,174 (80.1)	Reference	–
GA at delivery (weeks)^a^, Mean (sd)	34.7 (5.7)	38.8 (2.3)	1.28 (1.27, 1.30)	< .0001
Preterm, N (%)	364 (48.0)	2871 (9.5)	7.91 (6.92, 9.04)	< .0001
Multiple, N (%)	75 (9.9)	497 (1.6)	5.83 (4.51, 7.54)	< .0001
Essential newborn care				
Baby placed on mother's chest, N (%)	65 (8.8)	7258 (24.1)	0.28 (0.13, 0.57)	0.0006
Baby bathed within 6 h, N (%)	3 (0.4)	115 (0.4)	1.07 (0.52, 2.24)	0.8480
Breastfeeding initiation within 1 h, N (%)	128 (17.5)	21,114 (70.0)	0.08 (0.06, 0.11)	< .0001
Newborn treatment				
Bag and mask resuscitation, N (%)	379 (50.3)	1018 (3.4)	21.88 (18.48, 25.90)	< .0001
Antibiotics, N (%)	357 (47.3)	1837 (6.1)	11.81 (9.67, 14.42)	< .0001
Oxygen, N (%)	420 (55.6)	1615 (5.4)	18.26 (14.89, 22.39)	< .0001
CPAP, N (%)	147 (19.5)	434 (1.4)	13.61 (9.77, 18.98)	< .0001
Mechanical ventilation, N (%)	121 (16.0)	153 (0.5)	21.65 (17.00, 27.58)	< .0001

^a^Relative risk for a 1-week decrease in gestational age at delivery

^b^Relative risks and p-values are obtained from log Binomial models as a function of each individual neonatal characteristic using generalized estimating equations to account for the correlation of outcomes within cluster

Preterm births were also at higher risk for 28-day mortality with a RR of 7.9 (95% CI 6.9, 9.0) compared to infants ≥ 37 weeks. Additionally, a one-week decrease in gestational age at delivery was associated with a higher risk of mortality with a RR of 1.3 (95% CI 1.3, 1.3). Infants of multiple births had a RR of 5.8 (95% CI 4.5, 7.5) for 28-day neonatal mortality compared to singletons.

We evaluated three essential newborn care (ENC) practices. Of these, the use of both skin-to-skin contact after birth and early breastfeeding were associated with a decreased risk of 28-day neonatal mortality with a RR 0.3 (95% CI 0.1, 0.6) and RR 0.1 (95% CI 0.1, 0.1) respectively. Delayed bathing was not statistically associated with mortality risk (RR 1.1, 95% CI 0.5, 2.2).

Among infants who died by day 28, about half (50.3%) had received bag and mask ventilation at birth, less than half (47.3%) had received antibiotics, 55.6% received oxygen, 19.5% received continuous positive airway pressure (CPAP), and 16.0% were mechanically ventilated. In contrast, those alive at day 28, only 3.4% received bag and mask ventilation, 6.1% received antibiotics, 5.4% received oxygen, 1.4% received CPAP and 0.5% were mechanically ventilated. All differences were statistically significant.

**Fig. 3:**
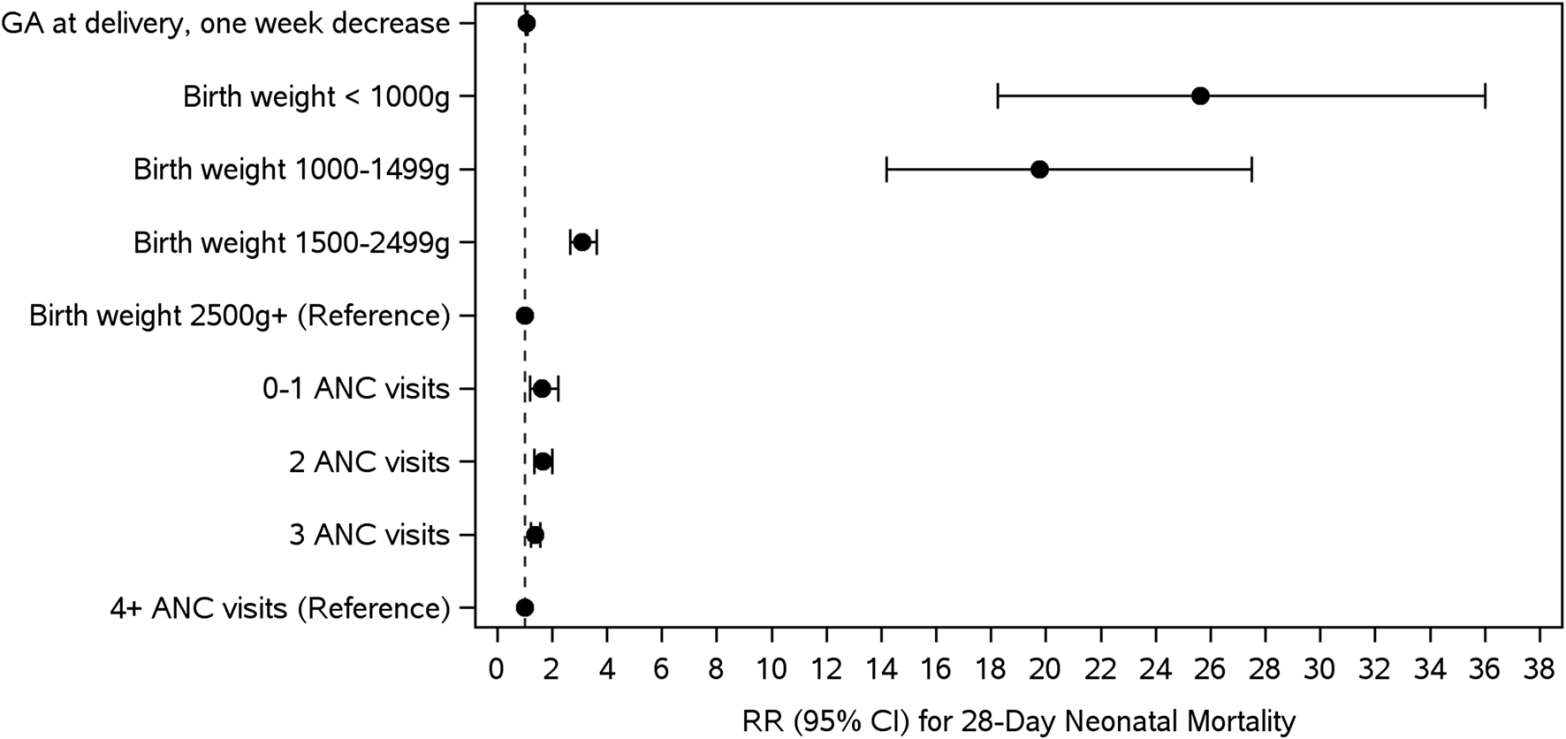
Predictive model for 28-day neonatal mortality

Finally, we developed a model to predict neonatal mortality by day 28 which evaluated the maternal and neonatal characteristics that had a statistically significant (p < 0.05) association with 28-day morality in the univariate models (Fig. 3). Neonatal treatments and interventions were excluded from these models as their occurrence is likely to be impacted by underlying risk factors as opposed to being primary risk factors themselves. Additionally, maternal characteristics (i.e., BMI) were excluded due to their lack of significant association with mortality. Thus, the final characteristics included in the predictive model were gestational age at delivery, birth weight, and the number of antenatal care visits. The marginal R^2^ value for this final model is 0.223 indicating that approximately 22% of the variability in mortality is explained by the included potential risk factors [16]. Low birth weight was the factor most predictive of 28-day neonatal mortality with adjusted relative risks as follows: infants born weighing < 1000 g RR was 25.6 (95%CI 18.3, 36.0), for 1000-1499 g infants the RR was 19.8 (95% CI 14.2, 27.5) and for 1500–2499 g infants the RR was 3.1 (95% CI 2.7, 3.6) (Fig. 3). Having fewer than four ANC visits also was predictive of 28-day neonatal mortality with the RR for having 0–1 visits most predictive (RR 1.6, 95% CI 1.2, 2.2). Lastly, the risk of 28-day mortality increases for each one-week decrease in gestational age at delivery (RR 1.1, 95% CI 1.1, 1.1).

## Discussion

This study of more than 30,000 births in rural India, collected from a population-based prospective registry, found that prematurity/low birthweight was the largest cause of death. This finding is consistent with a recent national study from India that found that prematurity contributed to 27% of under-five mortality [3]. In our study, neonatal infection and birth asphyxia were also important contributors to neonatal mortality. Among all live births, a birth weight < 1500 g had the largest association with risk of 28-day neonatal death. Other factors associated with increased risks of neonatal mortality included low levels of maternal education, high and low parity, and fewer ANC visits.

The strengths of this study included the prospective enrolment of pregnant women with a 99% follow-up through 28-days post-delivery. Study data were collected by trained study staff using a common protocol. A common methodology was used to assign cause of death using a prospectively designed algorithm. An important limitation of the study was our ability to interpret the ENC interventions and their impact on risk of mortality. Because the status of the baby may influence the likelihood of the infant to receive both ENC and other interventions, there was an inherent bias in the association of the intervention to mortality risk. That said, it was interesting to note that only about half of the infants who died had received the basic treatments of antibiotics or oxygen prior to their death, despite the majority now being delivered within the formal health system.

In conclusion, the study results point to the important association between prematurity as well as low-birth weight and 28-day neonatal mortality in India. We also noted that although the majority of births occur within health facilities, a relatively low number of infants who died received life-saving interventions. Further efforts to understand the impact of care on newborn outcomes are needed.

## Data Availability

Data from the study will be available at the NICHD data repository (N-DASH): https://dash.nichd.nih.gov/

## Abbreviations

ANC: Antenatal Care
BMI: Body Mass Index
COD: Cause of death
MNHR: Maternal Newborn Health Registry
RA: Registry Administrator
